# From Mutation to Manifestation: Evaluation of a *PKLR* Gene Truncation Caused by Exon Skipping in a Schnauzer Terrier

**DOI:** 10.3390/ani15243634

**Published:** 2025-12-17

**Authors:** Tzu Yi Ma, Chih Jung Kuo, Pin Chen Liu

**Affiliations:** 1 Department of Veterinary Medicine, College of Veterinary Medicine, National Chung Hsing University, Taichung 40227, Taiwan; d110038002@mail.nchu.edu.tw (T.Y.M.); ck476@nchu.edu.tw (C.J.K.); 2 Veterinary Medical Teaching Hospital, College of Veterinary Medicine, National Chung Hsing University, Taichung 40227, Taiwan

**Keywords:** *PLKR* gene mutation, exon skipping, severe congenital anemia

## Abstract

A five-month-old, intact, female Miniature Schnauzer Terrier presented with persistent severe hemolytic anemia following an initial infection with *Babesia gibsoni* and *B. vogeli.* Despite treatment, severe regenerative anemia persisted, and the patient was unresponsive to antibiotics, as well as antiprotozoal and immunosuppressive agents. Subsequent laboratory tests and diagnostic imaging ruled out persistent hemiparasitic infections, immune-mediated diseases, or neoplasia. Genomic DNA and cDNA sequencing identified a point mutation in exon 8 (g.4978G>T) that introduced a premature termination codon, leading to exon 8 skipping and a single-nucleotide deletion at the exon 7–intron 7 boundary (c.966delG) during splicing. A 151 bp deletion in the coding region of the patient’s *PKLR* cDNA was subsequently detected, which ultimately resulted in pyruvate kinase deficiency. This missplicing results in a premature stop codon and disrupts PKLR tetramer formation owing to the partial loss of domain A and complete loss of domain C. Enzyme activity assays confirmed a complete loss of function in the mutant PKLR protein compared to the wild-type, supporting the causal role of this deletion in non-spherocytic hemolytic anemia. This is the first report as per our knowledge documenting truncated PKLR variant in a dog, and notably, the first such case in a Miniature Schnauzer breed.

## Simple Summary

Pyruvate kinase deficiency (PKD) is a rare inherited disorder that causes chronic hemolytic anemia in humans and animals. Here, we report the first case of PKD in a Schnauzer Terrier in which a point mutation in exon 8 is associated with a splice defect that leads to a truncated PKLR protein. The affected dog showed severe anemia and nearly absent enzyme activity. Transcript analysis confirmed the loss of multiple exons and disruption of protein function. These findings highlight the importance of genetic testing for unexplained anemia in dogs and expand our understanding of the molecular spectrum of PKD mutations in veterinary medicine.

## 1. Introduction

Pyruvate kinase deficiency (PKD) is a glycolytic enzyme disorder and the most common hereditary non-spherocytic hemolytic anemia in humans, dogs, and cats. Mammals possess two distinct pyruvate kinase (PK) genes: *PKM*, which is expressed in muscle and various other tissues, and *PKLR*, which encodes the liver (L-type) and red blood cell (R-type) isoenzymes [1]. PK plays a critical role in erythrocyte metabolism by catalyzing the irreversible transphosphorylation of phosphoenolpyruvate (PEP) and adenosine diphosphate (ADP) into pyruvate and adenosine triphosphate (ATP). Consequently, PKD leads to ATP depletion, which ultimately affects cell viability. In humans, over 300 mutations in the *PKLR* gene have been reported, with missense variants being the most common; non-missense (NM) mutations such as insertions, deletions, and splice-site changes have also been documented [2,3]. The canine *PKLR* gene (Gene ID: 490425), located on chromosome 7, is tissue-specifically spliced into R-PK in late erythroid precursors and L-PK in hepatocytes. Most canine studies focused on breed-specific missense mutations. For instance, a c.433delC deletion causes PKD in Basenjis, whereas a 6 bp insertion in exon 10 is responsible for PKD in West Highland White and Cairn Terriers [4,5]. Other reported variants include c.848T>C in Pugs, c.994G>A in Beagles, and c.799C>T in Labrador Retrievers [5]. The prevalence of these mutations in cats varies among breeds. Abyssinian and Somali cats from the United States, Australia, and Germany show carrier frequencies of up to 16%, whereas lower rates (3–4%) are observed in domestic cats in Japan [5,6,7].

The wide variability in the clinical expression of PKD, along with technical difficulties and limited availability of PK enzyme assays, has led to its underdiagnosis [8]. Consequently, many affected individuals or animals may live with compensated anemia [9]. Patients harboring homozygous NM mutations in the *PKLR* gene tend to exhibit more severe clinical phenotypes. However, enzyme activity levels do not consistently correlate with hemolysis severity [1]. Mutations have also been identified in introns, promoter/enhancer elements, consensus splice sites, and the 5′ untranslated region, thus affecting regulatory domains such as the GATA1 binding site or even deleting entire exons, including exon 11. Among all the reported mutations, single-nucleotide substitutions are the most common (up to 72%), followed by small insertions and deletions (~13%).

Patients with PKD commonly develop complications, such as endocrinopathies, leg ulcers, bone disease, pulmonary hypertension, gallstones, and iron overload. Notably, progressive osteosclerosis of the long bones has been observed exclusively in dogs with PKD, and has not been reported in other species [5]. Iron overload in patients with PKD can lead to serious complications, including liver cirrhosis, cardiomyopathy, arrhythmia, sudden cardiac death, and endocrine dysfunction, regardless of transfusion history [9]. The erythroferrone–hepcidin axis plays a pivotal role in iron overload. Hypoxia induced by chronic hemolytic anemia stimulates erythropoietin, which, in turn, promotes erythroferrone production, suppresses hepcidin production, and increases iron absorption [10]. Management options include red blood cell (RBC) transfusion, iron chelation therapy, and splenectomy. Hematopoietic stem cell transplantation has been attempted in only a limited number of cases [2]. However, bone marrow transplantation in a canine model [11] and splenectomy in Basenjis [12] failed to resolve hematologic abnormalities. Mitapivat, a novel oral PK activator currently under clinical investigation, improves hemoglobin levels and reduces hemolysis markers in patients carrying at least one *PKLR* missense mutation [9].

The biophysical, biochemical, and kinetic properties of PK, particularly their allosteric regulation by metabolic effectors, have been extensively investigated. PK exists in two conformations, an active R-state and an inactive T-state, and transitions based on effector binding. PEP, fructose 1,6-bisphosphate (FBP), and low pH promote the R state, whereas ATP, alanine, and phenylalanine inhibit it. Additional regulation occurs through proteolysis, N-terminal phosphorylation, and hormone-induced dimerization. Structural studies revealed the key residues and interactions involved in this transition [13,14,15,16]. To investigate the effects of mutations on PK structure and function, we cloned, expressed, purified, and characterized the full-length recombinant canine R-type PK. Simultaneously, a mutant PK protein was produced, representing the first characterized recombinant mutant of canine PK.

Here, we present the first fully documented case of PKD in a Miniature Schnauzer Terrier, including long-term clinical follow-up, and the first reported necropsy findings in a dog with PKD. Genomic DNA and cDNA sequencing identified a point mutation in exon 8 (g.4978G>T) that introduced a premature stop codon, resulting in missplicing characterized by the loss of a single nucleotide at the end of exon 7 and skipping of exon 8. This missplicing event produced a premature stop codon that led to the partial loss of domain A and complete loss of domain C of PKLR, thereby disrupting tetramer formation. An enzyme kinetic assay confirmed that this mutation resulted in complete loss of enzymatic function. This comprehensive investigation, from mutation to clinical manifestations, offers new insights into the molecular pathogenesis and progression of PKD in dogs, with broader implications for similar metabolic disorders across species. Given the clinical severity and novelty of the structural variant, we hypothesized that the missplicing abolishes the enzyme activity by impairing domain C-dependent tetramer formation. To test this, we cloned and expressed both wild-type and mutant canine PK and compared their enzymatic activity and structural stability.

## 2. Materials and Methods

### 2.1. Sample Collection and Clinical Evaluation

A five-month-old, intact female Miniature Schnauzer was referred to the National Chung Hsing University Medical Teaching Hospital for evaluation of severe anemia (hematocrit: 9.1%) identified during routine health screening. The referring clinic suspected *Babesia gibsoni* infection. The only clinical sign reported by the owner is lethargy. On physical examination, the patient was tachycardic, with pale mucous membranes and a systolic heart murmur. Complete blood count revealed marked regenerative anemia with mild leukocytosis and lymphocytosis. Serum biochemistry showed mild hyperbilirubinemia (see Appendix A for a summary of clinicopathological data, including serum biochemistry, complete blood count, and urinalysis). Abdominal radiography revealed splenomegaly. Polymerase chain reaction (PCR) testing confirmed coinfection with *B. gibsoni* and *B. vogeli*, whereas *Ehrlichia canis*, *Anaplasma platys*, and *Mycoplasma* spp. tested negative. The cold-reactive direct Coombs test yielded positive results. The patient was hospitalized and treated with whole blood transfusion, diphenhydramine, corticosteroids, and a combination of antiprotozoal agents, including clindamycin, diminazene, and imidocarb. By day 3, the hematocrit had improved to 32.3%, and the patient was discharged in a stable condition.

Mild anemia and hyperbilirubinemia persisted at the follow-up evaluation on days 10 and 30, although PCR results were negative. Coombs’ test results were positive. Despite persistent hematological abnormalities, the patient exhibited improved energy, appetite, and behavior. The owner discontinued further follow-up. On day 82, the dog sustained a femoral fracture due to the trauma and underwent surgical repair after a second blood transfusion. No additional treatments were administered because the patient remained clinically stable. Regular follow-ups every six months revealed persistent regenerative anemia and mild hyperbilirubinemia (Table 1) without clinical deterioration. On day 720, the dog was euthanized due to severe sepsis associated with pyometra. Histopathological examination was performed after autopsy.

### 2.2. RNA and Genomic DNA Extraction and cDNA Synthesis

Total RNA and genomic DNA were extracted from the cephalic veins of both the patient and a healthy Schnauzer (male, 2 years old; reference dog) in ethylenediaminetetraacetic acid tubes using the QIAamp RNA Blood Mini Kit and the QIAamp DNA Blood Kit (Qiagen, Hilden, Germany), respectively. cDNA was synthesized from the RNA template using the MMLV Reverse Transcription Kit (Millipore Sigma, St. Louis, MO, USA) according to the manufacturer’s instructions. The forward (5′-ACCACAGTCCATGCCATCAC-3′) and reverse primers (5′-TCCACCACCCTGTTGCTGTA-3′) were used to amplify *PK* gene using PCR. Genomic DNA fragments containing exons 7–10 were amplified using TaKaRa Ex Taq polymerase (TaKaRa, Dalian, China) with the forward primer (5′-CTTGGGTTCCTGACCTCTG-3′) and the reverse primer (5′-GTCTTCATGCCTGATAACAAGAC-3′). The amplified products were analyzed on a 1.2% agarose gel and sequenced (Genomics BioSci & Tech Co., Ltd., New Taipei City, Taiwan). cDNA sequences were translated into amino acid sequences using the ExPASy server translation tool https://web.expasy.org/translate/ (accessed on 4 March 2025). Nucleotide and protein sequences were assembled and analyzed using BioEdit Version 7.2.5.

### 2.3. Gene Cloning and Recombinant Protein Expression

The construction of *PK* plasmid was introduced using the forward primer 5′-GGAATTCCCATGGATGTCCAGTCAGGAGAACATACAACC-3′ and reverse primer 5′-CCGCTCGAGTCAGGATATGCTGAGCACTCGCATG-3′, which contain NcoI and XhoI cleavage sequences (underlined), respectively. The PCR product of the *PK* gene was ligated into the pET32a vector (Novagen, Madison, WI, USA), which encodes a thioredoxin fusion partner and a His-tag at the N-terminus of the target protein. The recombinant *PK* plasmid was then used to transform *Escherichia coli* DH5α competent cells, and the transformed cells were streaked on a Luria–Bertani (LB) agar plate containing 100 mg/mL ampicillin. Ampicillin-resistant colonies were selected from the agar plates and sequenced to detect the *PK* gene. The correct construct was subsequently transformed into *E. coli* BL21(DE3) cells for protein expression. For protein overexpression, 5 mL of an overnight culture of a single transformant was used to inoculate 500 mL of fresh LB medium containing 100 mg/mL ampicillin. The cells were grown to an optical density at 600 nm of 0.6 and induced with 1 mM isopropyl-β-thiogalactopyranoside. After 4–5 h of induction at 37 °C, the cells were harvested by centrifugation at 7000× *g* for 15 min. Recombinant PK was purified at 4 °C. The cell paste obtained from 1 L of cell culture was suspended in 40 mL lysis buffer containing 25 mM Tris-HCl (pH 7.5) and 150 mM NaCl. A French press instrument (AIM-AMINCO Spectronic Instruments, Cambridge Scientific Products, Watertown, MA, USA) was used to disrupt the cells at 12,000 psi. The lysis solution was centrifuged, and the debris was discarded. The cell extract was loaded onto a 10 mL Ni-NTA column equilibrated with the same buffer containing 5 mM imidazole. The column was washed with 5 mM imidazole, followed by washing with 30 mM imidazole-containing buffer. His-tagged PK was eluted with a buffer containing 300 mM imidazole. The protein solution was dialyzed twice with 1 L of lysis buffer. The protein concentrations used in the experiments were determined based on the absorbance at 280 nm. Protein purity was assessed using sodium dodecyl sulfate–polyacrylamide gel electrophoresis (SDS-PAGE). For Western blot analysis, proteins were transferred onto polyvinylidene difluoride membranes (Merck Millipore, Darmstadt, Germany). The membrane was blocked with 3% bovine serum albumin (BSA) in PBS containing 0.1% *v*/*v* Tween 20 (PBST) for 1 h at 37 °C. The membrane was washed with PBST on a shaker three times and incubated with 1.0 μg/mL polyclonal human PKLR antibody (Novus Biologicals, Centennial, CO, USA) in PBST containing 1% BSA for 1 h at 37 °C. The membranes were then washed with PBST on a shaker three times and incubated with mouse anti-rabbit IgG linked with horseradish peroxidase diluted (1:2000) in PBST containing 5% BSA for 1 h at 37 °C. The secondary antibody was discarded and the membranes were washed three times with PBST on a shaker. The 3,3′,5,5′-tetramethylbenzidine liquid membrane substrate (Avantor, Inc., Radnor, PA, USA) was added to the membrane, and the blots were imaged using the Bio-Rad Universal Hood Ii Molecular imager (Bio-Rad Laboratories, Inc., Hercules, CA, USA).

### 2.4. Enzyme Activity Assay

The enzyme activity of recombinant PK (rPK) from both the patient and the reference dog was assessed by coupling pyruvate production with reduced nicotinamide adenine dinucleotide (NADH) oxidation using lactate dehydrogenase (LDH), according to the method recommended by the International Committee for Standardization in Hematology [17]. The standard reaction mixture contained 100 mM Tris-HCl (pH 8.0), 100 mM KCl, 10 mM MgCl_2_, 0.5 mM EDTA, 0.2 mM NADH, 4U LDH, 10 mM PEP, and 1.5 mM ADP in a final volume of 100 μL in a microplate. Reaction was initiated by adding 1 μM rPK at 37 °C. NADH consumption was monitored by measuring the absorbance at 340 nm using a UV/Vis microplate reader (SPECTROstar Nano, BMG LABTECH, Ortenberg, Germany). All assays were performed in triplicate.

## 3. Results

### 3.1. Histopathological Analysis

**Figure 1:**
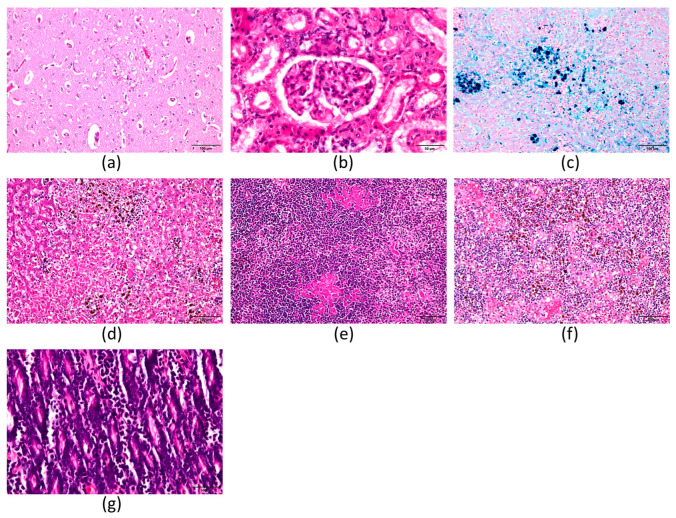
Histopathological and special staining findings in multiple organs from PKD-affected dog. (**a**) Histopathological evidence of chronic cerebral inflammation in a PKLR-deficient dog. Representative hematoxylin and eosin (H&E)–stained sections of the brain reveal multifocal lymphoplasmacytic meningitis characterized by perivascular and leptomeningeal infiltration of lymphocytes and plasma cells, with numerous intracytoplasmic eosinophilic granules. The adjacent parenchyma shows rarefaction and mild gliosis. These findings are consistent with chronic meningoencephalitis. Combined with the systemic hemosiderin accumulation identified in other organs, the cerebral lesions indicate a prolonged and severe inflammatory process that may have contributed to neurological dysfunction observed in the terminal phase (H&E, ×200). (**b**) Liver: Multifocal to coalescing hepatocellular necrosis with infiltration of lymphocytes and numerous pigment-laden macrophages in the necrotic foci, portal tracts, and perivascular regions. Hepatocytes are shrunken with a reticular cytoplasmic pattern, and abundant yellow-brown pigment granules are present. Kupffer cells contain large amounts of yellow-brown pigment. (H&E, ×200) (**c**) Spleen: The red pulp contains megakaryocytes and numerous nucleated erythrocytes, with pigment-laden macrophages containing yellow-brown granules scattered within the parenchyma. (H&E, ×200) (**d**) Lymph node: Megakaryocytes are present along with numerous pigment-laden macrophages containing yellow-brown granules. (H&E, ×200) (**e**) Intestine: In the lamina propria of the duodenum, jejunum, and ileum, inflammatory cell infiltration predominantly composed of plasma cells is observed, accompanied by fewer neutrophils and eosinophils. (H&E, ×400). (**f**) Kidney: Focal necrosis of renal tubular epithelial cells with mild neutrophilic infiltration. In the cortex, renal tubular epithelial cells contain small amounts of yellow-brown pigment, with cytoplasm appearing eosinophilic and granular. Some tubules are dilated, and their lumina contain eosinophilic material. (H&E, ×400) (**g**) Liver, Prussian blue staining: Iron-positive reactions are observed in the cytoplasm of hepatocytes, pigment-laden macrophages, and Kupffer cells. (Prussian blue, ×400).

Necropsy revealed severe systemic hemochromatosis with marked hepatosplenomegaly. Histologically, the brain exhibited chronic lymphoplasmacytic meningoen-cephalitis with prominent perivascular and leptomeningeal cuffs, many plasma cells containing eosinophilic granules, and mild parenchymal gliosis (Figure 1a); the liver showed multifocal to coalescing hepatocellular coagulative necrosis with shrunken hepatocytes, reticular cytoplasm, and abundant yellow-brown pigment in hepatocytes, Kupffer cells, and macrophages (Figure 1b); the spleen displayed marked extramedullary hematopoiesis with numerous megakaryocytes, erythroid precursors, and pigment-laden macrophages (Figure 1c); lymph nodes similarly contained prominent extramedullary hematopoiesis and heavy hemosiderin accumulation (Figure 1d); the small intestine demonstrated moderate to marked plasmacytic infiltration of the lamina propria with fewer neutrophils and eosinophils (Figure 1e); the kidneys revealed focal tubular epi-thelial necrosis, mild neutrophilic infiltration, tubular dilation with proteinaceous casts, and fine yellow-brown pigment within tubular epithelium (Figure 1f); Prussian blue staining confirmed extensive iron deposition in hepatocytes, Kupffer cells, and macro-phages in the liver (Figure 1g). Severe fibrinosuppurative and plasmacytic endometritis with isolation of Proteus mirabilis were also noted. These findings are consistent with end-stage pyruvate kinase deficiency (PKD) in dogs, which is characterized by chronic hemolytic anemia, secondary hemochromatosis, widespread extramedullary hematopoiesis, and multisystemic inflammation, leading to terminal neurological dysfunction.

### 3.2. Identification of PKLR Mutation

**Figure 2:**
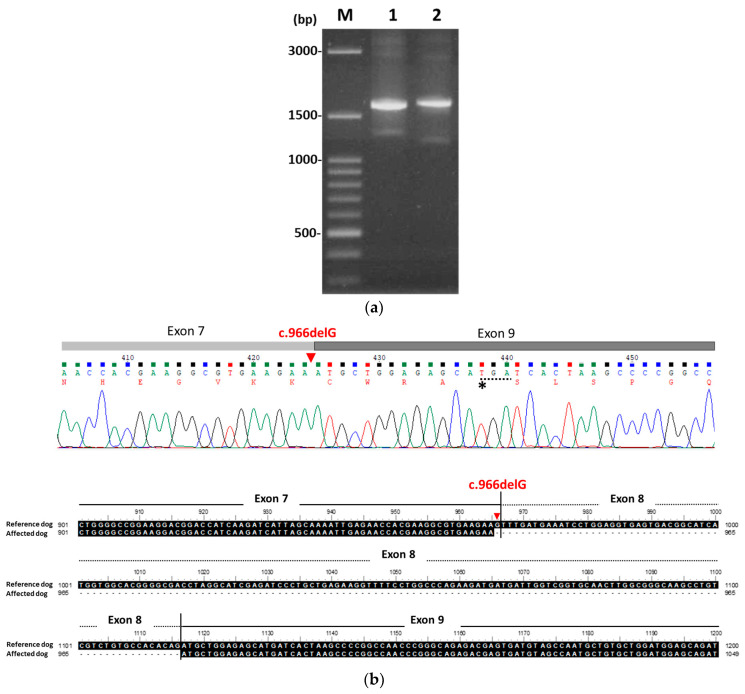

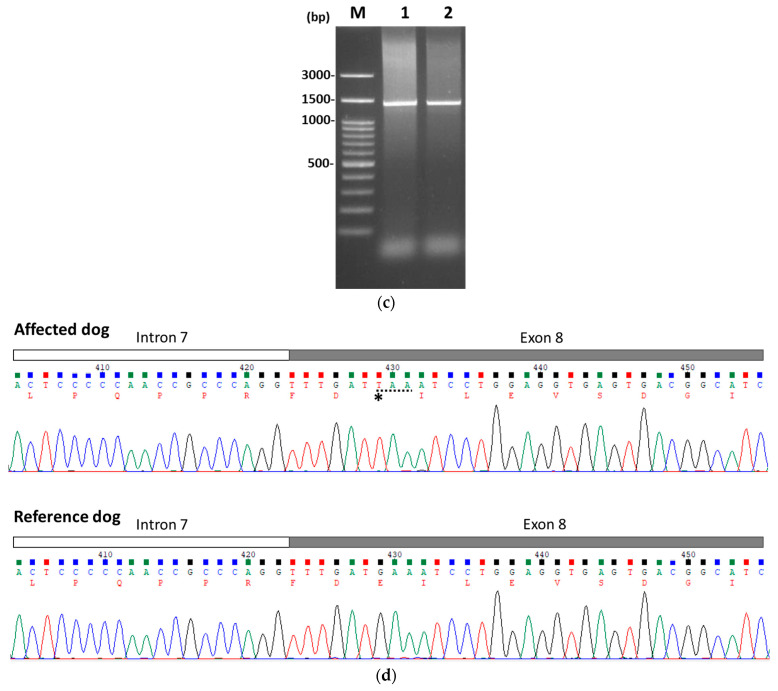
Sequence alignment reveals a deficiency in the *PKLR* gene of the affected dog. (**a**) The full-length *PKLR* cDNA amplified from the patient (lane 1) was markedly shorter than that from a reference dog (lane 2). (**b**) cDNA sequence analysis of the affected dog showed exon 8 skipping, along with a simultaneous single-base deletion (c.966delG, indicated with an inverted red triangle symbol) located at the last nucleotide of exon 7. This deletion caused a frameshift mutation, resulting in a premature stop codon (TGA, indicated with a star symbol) in exon 9 (upper panel). A comparison of the exon 7–9 cDNA sequences between the reference dog and the affected dog is shown in the lower panel, demonstrating a 151 bp deletion in the affected dog (lower panel). (**c**) The partial genomic DNA segment (exons 7–10) amplified from the patient (lane 1) and the reference dog (lane 2) yielded products of identical molecular weight (1419 bp). (**d**) Sequencing of the gene segment (exons 7–10) identified a homozygous point mutation (g.4978G>T, indicated with a star symbol) in exon 8 of the affected dog (upper panel), which was absent in the reference dog (lower panel). This exon 8 mutation introduced a premature stop codon (TAA) in the affected dog.

Full-length *PKLR* cDNA was amplified from the blood using gene-specific primers. The *PKLR* fragment in the affected dog was significantly shorter than that in the control dog (Figure 2a). Sequencing and alignment revealed a 151 bp deletion within the coding region of *PKLR* cDNA (Figure 2b, upper panel), confirming the molecular diagnosis of PKD. cDNA sequence analysis of the affected dog showed exon 8 skipping, along with a simultaneous single-base deletion (c.966delG) located at the last nucleotide of exon 7. This deletion caused a frameshift mutation resulting in a premature stop codon (TGA) in exon 9 (indicated by a star symbol). A comparison of the exons 7–9 cDNA sequences between the reference dog and the affected dog is shown in the lower panel of Figure 2b, demonstrating the missplicing observed in the affected dog. However, the genomic DNA segment spanning exons 7 to 10, amplified from both the affected dog and the reference dog, yielded products with identical molecular weights (1419 bp, Figure 2c). Gene sequencing analysis identified a homozygous point mutation (g.4978G>T) in exon 8 of the affected dog, resulting in a premature stop codon (TAA) (Figure 2d, indicated by a star symbol in the upper panel), when compared with the reference dog (lower panel). This premature termination codon may lead to nonsense-associated alternative splicing [18] and is predicted to result in a truncated protein lacking the entire C-terminal domain, which is essential for tetramer formation and enzyme activity.

### 3.3. Recombinant Protein Expression and Confirmation

**Figure 3:**
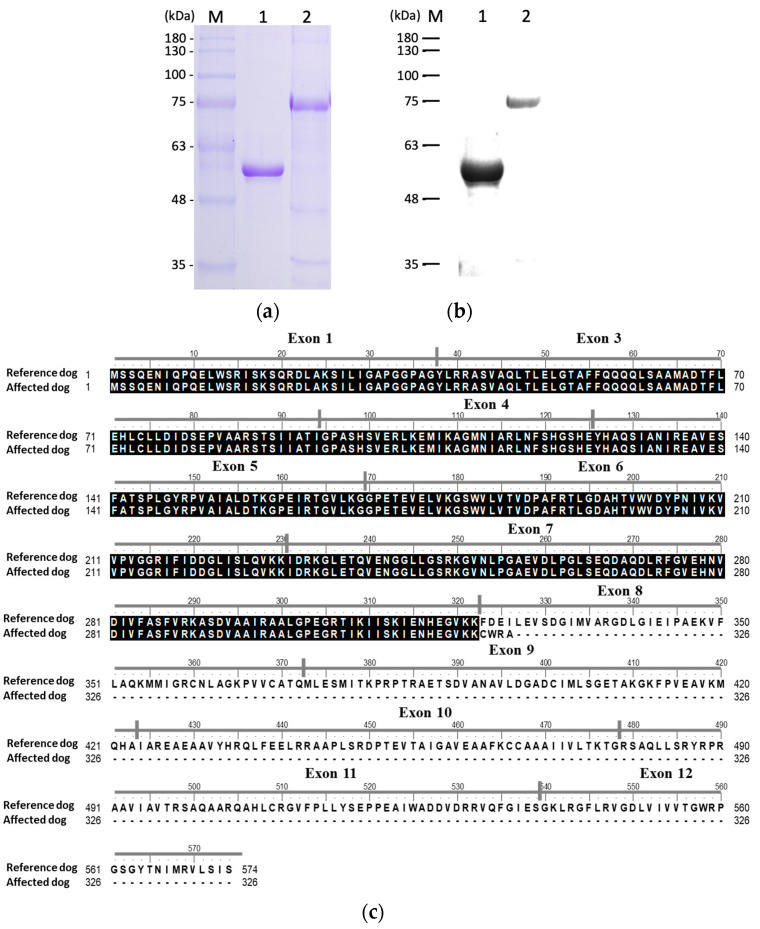
Expression and characterization of recombinant reference dog and mutant PKLR proteins. The coding sequences of *PKLR* derived from the affected dog (lane 1) and the reference dog (lane 2). (**a**) SDS-PAGE analysis reveals two distinct bands corresponding to the predicted molecular weights with the N-terminal fusion proteins: ~53.2 kDa and ~90 kDa, for the truncated mutant and the full-length wild-type PKLR proteins, respectively. (**b**) Western blot analysis using an anti–His-tag antibody confirmed the expression and integrity of both His-tagged recombinant proteins. The reduced molecular weight of the mutant protein reflects the loss of coding sequence caused by the exon splicing error, which introduced a premature stop codon and resulted in early translational termination, resulting in early translational termination and truncation of the C-terminal region of PKLR. These results validate the structural consequence of the identified mutation at the protein level. (**c**) PKLR protein sequence alignment between the reference dog and the affected dog showed that the affected dog’s protein terminates prematurely at residue 326 due to the 151 bp deletion causes a frameshift mutation, resulting in a downstream premature stop codon and truncation of the protein at residue 326. Compared with the full-length reference sequence, the predicted protein from the affected dog lacks the entire C-terminal region. This truncation is expected to eliminate the essential structural and functional domains of PKLR.

Full-length *PKLR* coding sequences from both patient and control dogs were cloned into pET32a expression vectors and expressed in *E. coli* BL21(DE3) cells. The SDS-PAGE analysis revealed two distinct bands: ~53.2 kDa for the truncated mutant PK protein and ~90 kDa for the wild-type PK protein, with the fusion proteins encoded by the expression vector (Figure 3a). Western blot analysis using anti-His-tag antibodies confirmed the identities of both recombinant proteins. (Figure 3b) The reduced molecular weight of the mutant protein was consistent with a premature stop codon introduced by missplicing in the *PKLR* transcript of the affected dog (Figure 3c).

### 3.4. Functional Characterization of Recombinant PK

**Figure 4:**
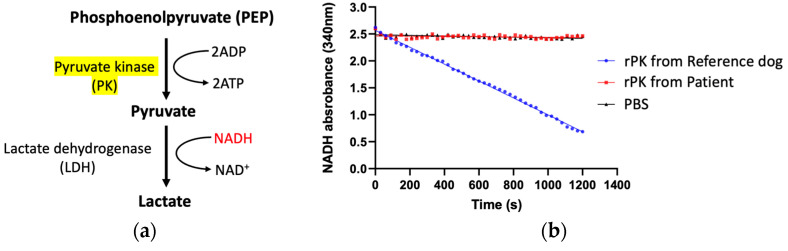
Kinetic analysis of recombinant pyruvate kinase (rPK) from the reference dog and patient. (**a**) Schematic of the lactate dehydrogenase (LDH)–coupled assay: Pyruvate kinase (PK) converts phosphoenolpyruvate to pyruvate with concomitant adenosine triphosphate generation. LDH then reduces pyruvate to lactate while oxidizing reduced nicotinamide adenine dinucleotide (NADH) to its oxidized form. The assay monitors the decrease in NADH at 340 nm, which is stoichiometric with PK activity. (**b**) Time course of NADH absorbance (340 nm) under identical assay conditions. rPK from the reference dog (blue) shows a progressive, approximately linear decline in NADH, thus indicating robust catalytic activity. rPK from the patient (red) remains essentially unchanged and overlaps the PBS control (black), which is consistent with immeasurable enzymatic activity.

The enzymatic activity of rPK was evaluated using an LDH-coupled spectrophotometric assay to measure NADH oxidation at 340 nm. Wild-type and mutant rPK were tested in parallel at equimolar concentrations. Wild-type rPK showed a time-dependent decrease in absorbance, consistent with the active conversion of PEP to pyruvate. This reaction profile was consistent across three independent replicates and exhibited linearity during the initial phase of the reaction, reflecting a reliable kinetic behavior. In contrast, mutant rPK displayed negligible activity. The absorbance at 340 nm remained unchanged throughout the assay and the result was indistinguishable from that of the negative control. The mutant enzyme also failed to respond to varying substrate concentrations, indicating complete loss of catalytic function. Quantification of the activity revealed a >95% reduction in the enzymatic activity of the mutant protein relative to that of the wild-type protein (Figure 4). SDS-PAGE confirmed the presence of the truncated protein, thus further corroborating the expression of an incomplete polypeptide.

## 4. Discussion

*PKLR* encodes both PK-R and PK-L isoenzymes via alternative promoter usage, with exon 1 specific to PK-R mRNA, exon 2 specific to PK-L mRNA, and shared exons 3–12 [2]. Although PKLR mutations can potentially affect both hepatic and erythrocyte isoenzymes, their clinical manifestations are predominantly confined to RBCs. This tissue-specific manifestation arises from the unique metabolic reliance of erythrocytes on glycolysis for ATP production, because they lack mitochondria. In contrast, hepatic cells can compensate for PKLR dysfunction through alternative metabolic pathways such as oxidative phosphorylation [19]. Mammalian PK functions as a homotetramer, with each monomer comprising distinct domains: domain (catalytic core), B domain (substrate binding), and C domain (allosteric regulation and subunit interactions). The C domain plays a vital role in tetramer stability and the binding of allosteric effectors such as FBP [14,20].

Premature stop codons (PTCs) alter pre-mRNA splicing through a mechanism known as nonsense-associated altered splicing. Several studies have shown that exons containing PTCs may be skipped due to the disruption of exon-splicing enhancers (ESEs) or changes in nuclear surveillance mechanisms. For example, in BRCA1, nonsense mutations disrupt the ESEs, leading to exon skipping [21]. In Neurofibromatosis type 1 (NF1), various PTCs such as W336X and Q315X cause partial skipping of exon 7 [22]. CRISPR-based studies have demonstrated that PTCs are key factors that drive exon skipping [23]. In the present study, the affected dog carried a point mutation (g.4978G>T) in exon 8 of *PKLR*, which generated a premature stop codon. This mutation simultaneously causes exon 8 skipping (150 bp) and a single-nucleotide deletion (1 bp) at the exon 7–intron 7 junction (c.966delG) at the mRNA level, resulting in a truncated PKLR protein.

Disruption of the C domain, as observed in this case, destabilizes the quaternary structure, resulting in complete loss of enzymatic function [14,16]. In our case, cDNA sequence analysis revealed missplicing in the *PKLR* transcript of the affected dog, resulting in the truncation of the C-terminal region. SDS-PAGE confirmed the expression of a low-molecular-weight protein consistent with truncation. Functional characterization using an LDH-coupled spectrophotometric assay demonstrated that the mutant rPK exhibited negligible catalytic activity under the assay conditions, with NADH absorbance levels remaining unchanged over time. This result was comparable to that of the negative control reactions lacking enzymes and indicated a >95% reduction in activity compared with wild-type rPK. No enzymatic response was observed across varying substrate concentrations, confirming the functional inactivation of the mutant PKLR protein. The observed enzymatic deficiency was consistent with the structural consequences of the 151 bp deletion, which removed domain C and part of domain A. Both domains are essential for enzymatic activity, subunit interaction, and structural stability. Structural studies have shown that disruption of domain C impairs tetramer assembly and allosteric regulation, leading to a complete loss of catalytic function [17,24]. Similar large deletions or premature truncations in PKLR have been reported in humans and cats and often correlate with more severe clinical phenotypes [20,25,26,27].

Canine PKD has traditionally been associated with missense mutations or small indels, such as an exonic deletion (c.433delC) in Basenjis and a 6 bp insertion in West Highland and Cairn Terriers [4,5]. To the best of our knowledge, this is the first study to report a *PKLR* splicing defect in a Miniature Schnauzer Terrier, expanding the known mutational spectrum of PKD. Similar large deletions have been documented in humans [27] and to a lesser extent in domestic cats [26]. The observed deletion resulted in a structural domain loss that was mechanistically similar to certain human PKLR variants associated with severe phenotypes, suggesting a conserved vulnerability in PK structure–function relationships across species [28].

**Figure 5:**
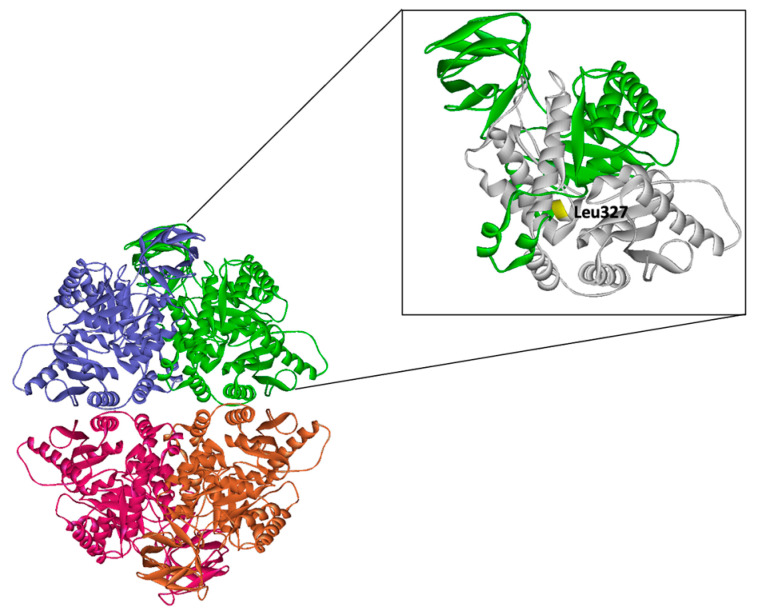
Structural position of Leu327 and predicted effect of the large deletion on PKLR quaternary structure. In the structural model, each monomer is colored purple, green, red, and orange, respectively. The deleted region (residues 327–574) is shaded in gray, whereas the remaining portion of the monomer is shown in green. The crystal structure of tetrameric PKLR is displayed, with individual subunits colored distinctly. The inset highlights Leu327 (shown in yellow), which is located at the C-terminal region of the A domain near the interface with the C domain. Although Leu327 is not directly involved in mitapivat binding, its hydrophobic side chain likely contributes to local structural stability at the interdomain boundary. The deletion removes the interdomain connection and the entire C domain and C-terminal α-helix, both of which are essential for C/C′ subunit interaction and tetramer formation. This structural disruption is predicted to impair tetramer assembly, thus rendering the mutant protein catalytically inactive and prone to misfolding or aggregation.

The diagnosis of PKD is complicated by factors such as recent blood transfusions, which can mask enzyme deficiencies caused by residual donor cells [29]. Consequently, consensus guidelines advocate a combined approach that uses both enzymatic assays and genetic analyses to improve diagnostic precision [1,30]. This combined strategy helps overcome the limitations of enzymatic testing, which may yield false-negative results due to increased reticulocytes, recent transfusions, or false-positive results in heterozygous carriers. Erythrocytes have a lifespan of approximately 120 days. Therefore, normal residual PK activity in donor cells can falsely elevate PK levels in patients with PKD who regularly receive blood transfusions. Direct comparisons with previous reports may be difficult due to the different testing methodologies [1]. Therefore, recent consensus recommendations suggest using both enzymatic testing and genetic analysis as complementary methods for a more precise diagnosis, even in the presence of a broad clinical phenotype and non-specific RBC morphology [1,30]. Although PK enzyme activity is helpful for diagnosis, it does not correlate with clinical severity and cannot predict clinical course [30]. Pathological manifestations typically arise when enzyme activity decreases to below 25% of the normal PK levels, with severe disease frequently linked to pronounced reticulocytosis [31]. This correlation aligns with the case of PKD Schnauzer, which showed very low levels of PK activity and a high degree of reticulocytosis. Few studies on enzyme activity in dogs with PKD have been reported, and the available literature dates back more than 20 years [4,12,29]. An international study found that patients with NM/NM mutations exhibited more severe phenotypes, including higher rates of iron overload, transfusions, splenectomy, and lower post-splenectomy hemoglobin levels than those with missense/NM or missense/missense PKLR mutations [1]. Leu327 is located in the C-terminal portion of the A domain near the interdomain boundary of the C domain. Although this residue does not directly participate in mitapivat binding, it likely plays a structural role in maintaining the integrity of the domain interface [9]. Its hydrophobic nature suggests that it contributes to the internal stability of the protein fold, and its loss may destabilize the local architecture required for proper C-domain positioning and drug-binding cavity formation (Figure 5) [9]. The large deletion spanning residues 327–574 abolishes the entire C domain and C-terminal α-helix of PKLR, which are essential for tetramerization through the C/C′ interface. Given that PKLR functions as a homotetramer and this quaternary structure is critical for catalytic activity, the mutant enzyme was structurally incapable of forming a stable tetramer (Figure 5). Consequently, they are expected to exist as misfolded monomers or inactive aggregates with severely compromised enzymatic function.

The autopsy findings further underscore the systemic effects of PKD. Significant iron overload was observed in multiple organs, including the spleen, liver, kidneys, and lungs, indicating chronic hemolysis and compensatory extramedullary hematopoiesis. Iron deposition in the central nervous system, which manifests as lymphoplasmacytic meningitis, is documented for the first time in a canine case of PKD. These novel autopsy findings suggest that iron overload contributes to neuroinflammatory processes and parallels observations in human PKD. Similar pathological features have been reported in human cases of PKD, in which chronic hemolysis and iron overload lead to hemosiderosis in various organs, including the liver, spleen, and central nervous system. Nagai et al. [32] described severe hemochromatosis involving multiple organs, suggesting parallels in systemic iron dysregulation between canine and human patients with PKD.

The current treatment strategies for PKD are primarily supportive and involve transfusion, splenectomy, and chelation therapy. In dogs, treatment is primarily supportive, including intermittent blood transfusions and splenectomy in some cases, although this has not consistently improved outcomes [12]. In contrast, human therapies have evolved to include iron chelation, bone marrow transplantation, and, most recently, pharmacological activation of PK using allosteric activators such as mitapivat [30]. However, such treatments require intact regulatory domains, rendering them ineffective in patients with large deletions involving the C domain, as in the current case. The deletion of the C domain likely renders such treatments ineffective, underscoring the need for alternative therapeutic approaches for patients with large structural mutations [33]. Finally, the patient was euthanized because of complications related to pyometra, a severe uterine infection that predisposes the patient to systemic sepsis and complicates surgical management. The increased cost and risk associated with ovariohysterectomy in these cases further highlight the challenges of managing PKD in dogs with comorbid conditions.

## 5. Conclusions

This study documents the first case of PKD in a Miniature Schnauzer Terrier resulting from missplicing in the *PKLR* mRNA spanning exons 8. This splicing defect leads to the production of a truncated, nonfunctional PK enzyme, causing severe clinical manifestations, including near-total loss of enzyme activity and significant iron overload in multiple organs. This case provides novel insights into the systemic effects of PKD, particularly its association with neuroinflammation caused by iron deposition. Given the loss of the C domain, treatments with PK activators (e.g., mitapivat) may be ineffective, underscoring the need for alternative therapeutic strategies. These findings highlighted the importance of integrating genomic, enzymatic, and pathological data to obtain a comprehensive understanding of PKD in companion animals.

## Figures and Tables

**Table 1:** Serial measurements of hematocrit (Hct), reticulocyte count, and total bilirubin (TBIL) over time.

Days	Hct (%) (37.3–61.7%)	Reticulocytes (×10^3^/μL) (10–110)	TBIL (mg/dL) (0.09–0.25 mg/dL)
0	10.6	–	–
1	9.1	516.1	0.55
2 *	32.3	639.5	0.42
10	26.8	177.2	0.22
30	24.9	747.9	0.37
38	22.5	717.0	0.28
84	17.6	679.8	–
86 *	32.3	554.6	–
88	30.5	245.8	–
103	26.9	560.9	–
118	24.4	656.5	0.70
233	16.0	877.9	0.74
360	15.8	1005.8	0.56
473	18.9	1144.8	0.60
609	21.7	1204.9	0.54

“–” indicates that data were not available or not measured on that date. “*” indicates that a whole blood transfusion had recently been administered. Complete blood counts were performed using an IDEXX ProCyte Dx* Hematology Analyzer (IDEXX Laboratories, Inc., Westbrook, ME, USA), and serum biochemistry tests were performed using a Beckman Coulter AU480 Automated Chemistry Analyzer (Beckman Coulter, Inc., Brea, CA, USA).

## Data Availability

The complete datasets used and/or analyzed in the current study are available from the corresponding author upon reasonable request.

## Abbreviations

ADP	Adenosine diphosphate
ATP	Adenosine triphosphate
ESEs	Exon-splicing enhancers
Hct	Hematocrit
LDH	Lactate dehydrogenase
NADH	Reduced nicotinamide adenine dinucleotide
NM	Non-missense
PBST	PBS containing 0.1% *v*/*v* Tween 20
PCR	Polymerase chain reaction
PEP	Phosphoenolpyruvate
PK	Pyruvate kinase
PKD	Pyruvate kinase deficiency
PTCs	Premature stop codons
rPK	Recombinant pyruvate kinase
SDS-PAGE	Sodium dodecyl sulfate–polyacrylamide gel electrophoresis
TBIL	Total bilirubin

## Appendix A

Summary of clinicopathological data, including serum biochemistry, complete blood count, and urinalysis.

**Table A1:** Serum Biochemistry.

Dyas	AST (U/L)	ALT (U/L)	ALP (U/L)	TBIL (mg/dL)	GLU (mg/dL)	BUN (mg/dL)	CREA (mg/dL)	TP (g/dL)	ALB (g/dL)	Na (mEq/L)	K (mEq/L)	Cl (mEq/L)	Ca (mEq/L)	Phos (mEq/L)	Mg (mEq/L)	CO_2_ (mmol/L)	OSMA	GLO (g/dL)	A/G	Chol (mEq/L)	GAP	TRIG (mg/dL)
0	-	-	-	-	-	-	-	-	-	-	-	-	-	-	-	-	-	-	-	-	-	-
1	111	108	150	0.55	103	28	0.9	7.1	3.2	142	4.1	110	-	-	-	-	308	3.9	0.8	-	-	-
2	-	-	-	0.42	-	-	-	-	-	-	-	-	-	-	-	-	-	-	-	-	-	-
10	-	-	-	0.22	122.0	-	-	5.9	3.1	-	-	-	-	-	-	-	-	2.8	1.1	-	-	-
30	-	-	-	0.37	136.0	-	-	6.2	3.5	-	-	-	-	-	-	-	-	2.7	1.3	-	-	-
38	-	-	-	0.28	-	-	-	-	-	-	-	-	-	-	-	-	-	-	-	-	-	-
82	-	-	-	-	-	-	-	-	-	-	-	-	-	-	-	-	-	-	-	-	-	-
84	-	103.0	125.0	-	-	-	-	-	-	-	-	-	-	-	-	-	-	-	-	-	-	-
118	-	-	-	0.7	-	-	-	-	-	-	-	-	-	-	-	-	-	-	-	-	-	-
233	30.0	21.0	68.0	0.74	133.0	10.0	0.7	6.6	2.9	144.0	4.6	111.0	10.3	4.5	2.3	25.7	308.0	-	-	177.0	11.9	-
360	35.0	27.0	47.0	0.56	118.0	19.0	0.6	7.1	3.3	145.0	4.2	113.0	10.3	4.9	1.9	19.2	312.0	3.8	0.9	161.0	17.0	-
473	30.0	20.0	52.0	0.6	120.0	18.0	0.5	7.1	3.1	146.0	4.7	113.0	9.9	3.8	1.9	23.7	314.0	4.0	0.8	199.0	14.0	52.0
609	30.0	20.0	58.0	0.54	117.0	19.0	0.5	7.1	3.1	145.0	4.5	110.0	10.5	3.2	2.0	26.1	312.0	4.0	0.8	181.0	13.4	152.0

“-” indicates the data was not available or not measured on that date. Referenc range: AST: 10–40 U/L; ALT: 7–56 U/L; ALP: 44–147 U/L; TBIL: 0.2–1.2 mg/dL; GLU: 70–105 mg/dL; BUN: 7–20 mg/dL; CREA = 0.4–1.3 mg/dL; Ca: 8.6–10.2 mg/dL; Phos: 2.5–4.5 mg/dL; Mg: 1.7–2.2 mg/dL;Na: 136–145 (mEq/dL); K:3.5–5.1 (mEq/dL); CO_2_: 22–29 mmol/L; Alb: 3.5–5 mEq/dL; GLO: 2.8–3.2 g/dL; Chol: 98–107 mEq/L; TRIG: <150 mg/dL. Serum biochemistry test was performed by Beckman Coulter Automated Chemistry Analyzer AU480.

**Table A2:** Complete Blood Counts.

Days	RBC (M/μL)	HCT (%)	HGB (g/dL)	MCV (fL)	MCH (pg)	MCHC (g/dL)	RDW (%)	%Retic	Retic (K/μL)	Retic-HGB (pg)	WBC (K/μL)	%Neu	%Lym	%Mono	%Eos	%Baso	PLT (K/μL)	MPV (fL)	PDW (fL)	PCT (%)
0	1.3	10.6	2.0	79.7	21.1	26.4	21.3	-	-	-	26.7	-	-	-	-	-	160.0	10.5	18.2	0.168
1	1.7	9.1	3.4	71.7	26.8	37.4	-	40.6	516.1	21.7	26.09	53.5	32.5	13.4	0.5	0.1	291.0	17.5	-	0.51
2	4.5	32.3	10.5	71.6	23.3	32.5	25	14.2	639.5	20.2	19.81	54.6	29.8	15.3	0.3	0.1	323.0	16.7	-	0.54
10	4.29	26.8	10.2	62.5	23.8	38.1	18.5	4.1	177.2	22.8	10.17	49.0	39.1	7.9	2.5	1.5	602.0	11.3	11.4	0.68
30	3.71	24.9	8.4	67.1	22.6	33.7	24.8	20.2	747.9	20.3	13.83	56.0	35.0	6.4	1.9	0.2	410.0	15.5	-	0.64
38	3.28	22.5	7.5	68.9	22.9	33.3	26.2	21.9	717.0	20.4	14.48	55.6	36.5	5.9	1.5	0.5	388.0	14.9	-	0.58
84	2.49	17.6	5.9	70.7	23.7	33.5	-	27.3	679.8	20.0	17.7	68.4	22.9	7.1	1.5	0.1	373.0	15.2	21.8	0.57
86	4.7	32.3	10.2	68.7	21.7	31.6	22.4	11.8	554.6	23.0	13.81	76.9	17.5	4.7	0.8	0.1	426.0	15.8	-	0.67
88	4.82	30.5	9.8	63.3	20.3	32.1	16.3	5.1	245.8	15.8	8.97	58.1	26.5	13.2	1.8	0.4	587.0	15.2	13.6	0.8
103	4.05	26.9	8.5	66.4	21.0	31.6	23	13.9	560.9	20.7	16.4	66.0	26.8	4.4	2.6	0.2	463.0	14.9	19.6	0.69
118	3.69	24.4	8.3	66.1	22.5	34.0	26.7	17.8	656.5	21.9	16.07	63.7	28.6	4.7	2.9	0.1	454.0	14.9	19.1	0.68
233	2.3	16.0	5.4	69.6	23.5	33.8	-	38.2	877.9	19.7	17.21	65.0	28.7	5.0	1.1	0.2	635.0	14.1	19	0.9
360	2.3	15.8	5.5	68.4	23.8	34.8	-	43.5	1005.8	20.2	16.99	56.5	36.6	5.2	1.6	0.1	647.0	13.7	17.3	0.89
473	2.43	18.9	5.7	77.8	23.5	30.2	-	47.1	1144.8	21.5	14.95	47.8	44.7	5.8	1.6	0.1	459.0	15.0	18.7	0.69
609	2.58	21.7	6.4	84.1	24.8	29.5	-	46.7	1204.9	25.4	16.63	47.5	45.7	5.7	1.0	0.2	537.0	15.2	20.9	0.82

“-” indicates the data was not available or not measured on that date. Reference range: RBC: 5.65–8.87 M/μL; HCT: 37.3–61.7%; HGB: 13.1–20.5 g/dL; MCV: 61.6–73.5 fL; MCH: 21.2–25.9 pg; MCHC: 32.0–27.9 g/dL; RDW: 12.9–17.6%; Retic: 10–110 K/μL; Retic-HGB: 22.3–29.6 pg; WBC: 4.9–17.6 K/μL; PLT: 148–484 K/μL; MPV: 7.3–11.5 fL; PDW: 9.1–16.7 fL; PCT: 0.11–0.46%. Complete blood count was performed by IDEXX ProCyte Dx* Hematology Analyzer.

**Table A3:** Urinalysis.

Days	Collection	Color	Clarity	Sp.Gr.	pH	Leu	Pro	Glu	Ket	Ubg (EU/dL)	Bil	BLD (Ery/μL)
118	Void	Amber	Slightly cloudy	-	7.0	25	Trace	-	-	1	1	-
360	Void	Amber	Cloudy	1.035	8.0	-	-	-	-	4	3	-
473	Void	Dark yellow	Slightly cloudy	1.042	6.5	-	-	-	-	1	3	-
609	Void	Orange-yellow	Cloudy	1.035	7.0	-	Trace	-	-	1	3	25

“-” indicates the data was not available or not measured on that date. Reference range: Specific gravity: 1.015–1.045; pH: 5.5–7.0; Protein: negative; Leu: negative; Pro: negtive; Glu: negative; Ket: negative; Ubg:0.1–1 EU/dL; Bil: negative-trace; BLD: negative; Urinalysis was performed by IDEXX VetLab UA Analyzer Notes: AST = aspartate aminotransferase; ALT = alanine aminotransferase; ALP = alkaline phosphatase; TBIL = total bilirubin; GLU = glucose; BUN = blood urea nitrogen; CREA = creatinine; Ca = calcium; Phos = phosphorus; Mg = magnesium; Co2 = bicarbonate; OSMA = osmolarity; GLO = globulin; A/G = albumin to globulin ratio; Chol = cholesterol; TRIG = triglyceride. RBC = red blood cells; HCT = hematocrit; HGB = hemoglobin; MCV = mean corpuscular volume; MCH = mean corpuscular hemoglobin; MCHC = mean corpuscular hemoglobin concentration; RDW = red cell distribution width; Retic =reticulocytes; PLT = platelets; MPV = mean platelet volume; PDW = platelet distribution width; PCT = plateletcri; Ubg = urobilinogen; Bil = bilirubin; BLD = blood; Leu = leukocytes; Glu = glucose; Ket = ketones; Sp.Gr. = specific gravity; pH = potential of hydrogen.
